# Clinical attachment loss in the use of adjunctive antimicrobial photodynamic therapy in Stages II-IV Grade C molar-incisor periodontitis: A systematic review and meta-analysis

**DOI:** 10.17305/bjbms.2022.7157

**Published:** 2022-05-29

**Authors:** Zahra Baghani, Samira Basir Shabestari, Malihe Karrabi

**Affiliations:** 1 Department of Periodontics, Faculty of Dentistry, Sabzevar University of Medical Sciences, Sabzevar, Iran; 2 Department of Oral Medicine, Firoozgar Hospital, Iran University of Medical Sciences, Tehran, Iran; 3 Department of Periodontics, Faculty of Dentistry, Sabzevar University of Medical Sciences, Sabzevar, Iran

**Keywords:** Aggressive periodontitis, attachment loss, photo chemotherapy, periodontal debridement, dental scaling, therapy

## Abstract

This systematic review and meta-analysis aimed to assess the extent of clinical attachment loss (CAL) as a clinical parameter in the efficacy of antimicrobial photodynamic therapy (aPDT) in non-surgical management of Stages II-IV Grade C molar-incisor pattern periodontitis. This review protocol was conducted in accordance with Preferred Reporting Items for Systematic Reviews and Meta-Analysis statements and is registered in PROSPERO (CRD42022321211). An electronic and manual search was conducted for relevant articles comparing the efficacy of aPDT versus scaling and root planning (SRP) alone or with amoxicillin/metronidazole (AMX/MET) published up until December 2021. The mean CAL, probing depth reduction, and BOP with a 95% confidence interval were pooled and compared between the two groups with CAL < and > 7 mm using a random-effect model after 3 and 6 months. To assess the heterogeneity of the findings, the I2 test was applied, and publication bias was evaluated by visual examination of the funnel plot symmetry. Analysis of nine studies indicated a significant difference in clinical attachment gain in patients with CAL > 7 mm between the aPDT group and the SRP alone (mean difference = 0.92, 95% CI = 0.01-1.84, *p* = 0.05) and SRP + AMX/MET (mean difference = 0.91, 95% CI = −0.14-1.68, *p* = 0.02) control groups. However, this difference was not significant in patients with CAL < 7 mm. Despite the limitations of the included studies, aPDT can be suggested to improve clinical parameters in Grade C molar-incisor pattern periodontitis with CAL > 7 mm. However, its application in milder cases requires further investigation.

## INTRODUCTION

Aggressive periodontitis (AgP) is an inflammatory disease characterized by rapid alveolar bone destruction and extensive clinical attachment loss (CAL) that occurs in response to polymicrobial factors and can be mild, moderate, or severe [1,2]. Although in the last world workshop on the classification of periodontal and peri-implant diseases, severity (Stages I-IV) or complexity and speed of progression were the basis of the new classification, Grade C represents the rapid rate of progression [3]. The speed of disease progression and the complications of management are due to the self-limiting nature of this disease and the impaired innate immunity [4]. Thus, AgP is different from chronic periodontitis (CP), and it is better assessed separately. The provision of prompt and efficient treatment with no side effects and long-term maintenance of the results are the challenges clinicians encounter in managing patients with AgP to prevent early tooth loss in young patients [5].

Non-surgical periodontal therapy is the treatment protocol primarily suggested in the management of AgP to transiently eliminate or decrease the count of microorganisms and improve the clinical parameters [6,7]. Systemic or local antibiotic therapy is another treatment the modality for the treatment of periodontitis. Despite the positive clinical results, this modality did not gain wide acceptance due to shortcomings such as the risk of antibiotic resistance, side effects of antibiotic therapy [8], difficult manipulation, risk of displacement in topical application, and the need for high patient cooperation [9]. Considering the drawbacks of the above-mentioned two modalities, antimicrobial photodynamic therapy (aPDT) was introduced as a novel alternative with the same advantages and no serious complications as an adjunct to mechanical treatments for periodontitis [10,11]. In aPDT, the chemical molecules of the photosensitizer are stimulated by a light source with a wavelength compatible with the absorption spectrum of the photosensitizer and produce reactive oxygen species. Such reactive species cause antibacterial reactions in more classes of microorganisms such as Gram-positive and Gram-negative bacteria [12], fungi [13], and viruses [14], which are safe, more acceptable as non-surgical methods of treatment, not toxic to the human tissues, and cannot cause bacterial resistance either [15-17]. Although according to the Specific Plaque theory (1977) [18], the oral disease could be initiated by a number of specific pathogens, Keystone theory (2012) [19] indicates that certain low-abundance microbial pathogens can cause inflammatory disease by increasing the quantity of the normal microbiota and by changing its composition. Therefore, it seems that the composition of the normal flora around bacterial niches of microorganisms that are affected by aPDT is not normal.

Extensive clinical studies on humans have reported the positive effect of aPDT on clinical parameters such as CAL, PPD, and bleeding on probing (BOP) and immunological parameters in patients with AgP compared with scaling and root planning (SRP). A reduction of orange and red complex species of microorganism and significantly lower mean levels of IL-1β in deep periodontal pockets were observed at a 3-month follow-up [20-24]. However, some studies could not confirm these effects with a high level of certainty [10,25,26]. On the other hand, some research has not definitively indicated positive effects of aPDT in comparison to SRP or AMX/MET. Thus, a conclusive result regarding the application of a PDT for the treatment of AgP has not been reached. One reason for not reaching a definite conclusion in this respect is the existing controversy regarding the effects of parameters such as frequency of aPDT sessions, type of photosensitizer, the effective energy density per square centimeter, and the efficacy of a PDT based on disease severity, which should be further elucidated. To achieve a reliable conclusion regarding the application of aPDT, several meta-analyses[24,27,28] evaluated the efficacy of a PDT along with SRP for the treatment of AgP and showed its comparable clinical efficacy to SRP + amoxicillin-metronidazole (AMX/MET). However, when compared with SRP alone, aPDT only caused significant improvement in deep pockets. However, limitations such as high variability in aPDT variables such as voltage, wavelength, and type of photosensitizer, and heterogeneity across the studies did not allow the study to reach a definite and reliable conclusion. A recent meta-analysis [28] on this topic evaluated five randomized clinical trials (RCTs) on the effect of aPDT on clinical parameters such as pocket depth, CAL, and bleeding on probing (BOP) compared with antibiotic therapy with AMX/MET after SRP in periodontitis patients. Despite the limitations of included studies and the high heterogeneity of the findings, the results indicated comparable efficacy of aPDT with systemic antibiotic therapy with AMX/MET in combination with mechanical debridement.

Overall, not assessing the effect of aPDT based on disease severity and evaluation of both AgP and CP as one entity (periodontitis) [28,29] can cause under- or over-treatment. Moreover, active disease parameters such as BOP and plaque score reflect the presence of active disease and the patient’s ability to perform proper plaque control and no severity of the disease. Hence, the CAL parameter is selected to estimate the extent of periodontal disease and destruction of the periodontium. Therefore, this systematic review and meta-analysis aimed to assess the effect of adjunctive aPDT on Stages II-IV Grade C molar-incisor pattern periodontitis patients with CAL < 7 mm and > 7 mm to prevent under- or over-treatment.

## MATERIALS AND METHODS

### PICO protocol and search strategy

This systematic review was conducted in accordance with the Preferred Reporting Items for Systematic Reviews and Meta-Analysis (PRISMA) Statement guidelines and Cochrane Collaboration recommendations. The systematic review protocol was registered in the Prospective Register of Systematic Reviews (PROSPERO); ref CRD 42022321211. The PICO components were as follows:

- Population: Patients diagnosed with Stages II-IV Grade C molar-incisor pattern periodontitis
- Intervention: aPDT along with SRP
- Comparison: SRP alone or along with antibiotic therapy with AMX/MET
- Outcome: CAL, PD, clinical attachment gain, and BOP
- Focused question: Can the extent of CAL affect the efficacy of aPDT as an adjunct to SRP or AMX/MET in Stages II-IV Grade C molar-incisor pattern periodontitis patients?

### Search strategy

The keywords were selected according to MeSH to determine the search strategy. The search algorithm was as follows:

“Early-Onset Periodontitis” [MeSH] OR “Juvenile Periodontitis” [MeSH] OR “Aggressive Periodontitis” [MeSH] OR “Periodontal disease” OR Periodontitis [MeSH] OR “Periodontal pockets” [MeSH] OR “Alveolar bone loss” OR “Attachment loss”

AND

“Non-surgical therapy” OR “Photochemotherapy” [MeSH] OR “Photodynamic therapy”

AND

“Scaling Root planing” OR “Dental Scaling” [MeSH] OR “Dental Root planing” OR “Periodontal debridement” [MeSH]

The keywords were searched electronically in Cochrane, Medline, and EMBASE databases and manually in the following journals: Journal of Periodontology, Journal of Clinical Periodontology, Journal of Periodontal Research, Laser in Medical Science, Periodontology 2000, and Photo diagnosis and Photodynamic Therapy.

**FIGURE 1:**
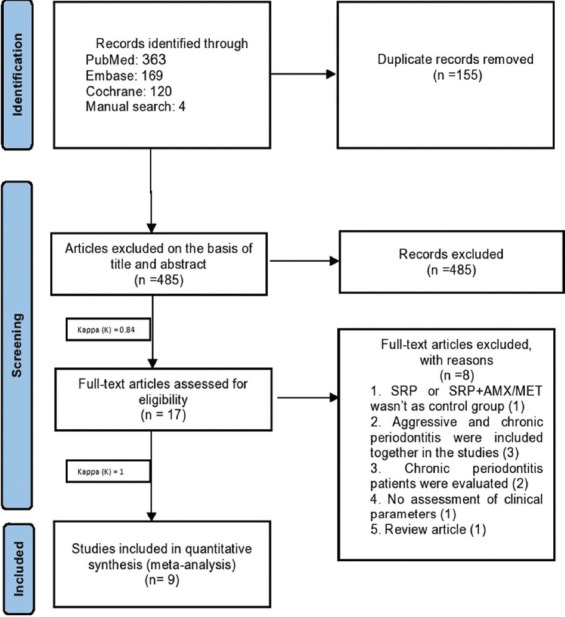
Flowchart of the search strategy. SRP: Scaling Root planning, AMX/MET: Amoxicillin/ Metronidazole.

A title/abstract search was conducted from the first record up until December 2021. The search was conducted by two blinded reviewers (ZB and SB), and inter-reviewer reliability analysis was also conducted [30]. The screening of titles/abstracts was performed to find eligible studies. The disagreements between the reviewers regarding the inclusion criteria were resolved by discussing a third reviewer (MK). Next, the full text of the eligible articles was read. Figure 1 shows the PRISMA flow diagram of the screening process.

### Eligibility criteria

#### Inclusion criteria

Studies that met the following inclusion criteria were included in the meta-analysis:

- Stages II-IV Grade C molar-incisor pattern periodontitis patients who met the 2017 diagnostic criteria for periodontal disease or AgP patients according to 1999 diagnostic criteria of periodontal disease
- Parallel or split-mouth RCTs
- Studies with a control group of SRP alone or SRP combined with antibiotic therapy with AMX/MET
- A PDT + SRP as the intervention group
- Assessment of CAL and PD
- Reporting the mean and standard deviation of the variables
- Minimum follow-up of 3 months
- No limitation with respect to the type of photosensitizer, voltage, or wavelength of light
- English articles.

#### Exclusion criteria

- Assessment of the efficacy of aPDT for the treatment of CP or studies that did not differentiate between AgP and CP patient
- *In vitro* studies
- Case reports, case series, systematic reviews, unpublished articles, letters to editors, and abstracts
- Presence of systemic diseases
- Smoker patients.

### Data extraction

After reading the full text of the articles, the review, quality assessment, and data extraction were performed by two independent reviewers. The second author (ZB) confirmed the accuracy of the extracted data, and the ambiguities were discussed with the third author (MK) until a consensus was reached. The extracted data included the first author’s name, country, age, gender and number of patients, follow-up time, laser parameters (voltage and wavelength), type of photosensitizer, frequency of treatment sessions, CAL, PD, and BOP parameters (as mean and standard deviation), and dosage, and duration of antibiotic therapy.

### Risk of bias (RoB)

According to the Cochrane Handbook for Systematic Reviews of Interventions, the Revised Cochrane RoB tool for randomized trials, version 2.0 (RoB 2) for each included study was independently assessed as follows by two of the authors (ZB and SB): This analysis was evaluated under the following headings:

1. Bias arising from the randomization process
2. Bias due to deviations from intended interventions
3. Bias due to missing outcome data
4. Bias in measurement of the outcome
5. Bias in the selection of the reported result.

The risk of bias was categorized as low, some concerns and high. Disagreements between the reviewers were resolved by consultation with a third author (MK).

### Data synthesis

Details of the studies extracted independently by the two reviewers (ZB and SB) were as follows: Age, gender, number of patients, diagnostic criteria for periodontitis, type of intervention, type of photosensitizer, laser parameters (wavelength, power, duration of radiation, and energy density), follow-up time, and clinical parameters (CAL, PD, and BOP). The extracted clinical findings included PD, CAL, and BOP in millimeters (mm) which were reported as mean and standard deviation, and were tabulated. Data were entered into RevMan version 5.0. The missing data necessitating contact with the corresponding author did not exist in any study.

### Meta-analysis

Some recent achievements indicated aPDT promotes additional positive clinical effects in deep pockets and attachment loss > 7 mm of Grade C, Stages III-IV periodontitis [20,23]. It seems attachment loss > 7 mm can be used as a classification basis. Thus, due to no evaluation of different stages of disease in RCTs, the included studies were categorized into two groups, with CAL < 7 mm and CAL > 7 mm. The effect of treatment was reported as a mean difference with a 95% confidence interval (CI). Furthermore, in some studies, SRP was performed along with antibiotic therapy for the control group. Hence, in order to increase the accuracy of the results, within-group analysis was also conducted in SRP + antibiotic therapy group and SRP alone at 3- and 6-month follow-ups. The random-effect model of RevMan version 5 was also used for data analysis at *P* < 0.05 level of significance. To detect statistical heterogeneity, forest plots were visually inspected through the presence of outlier studies. For the assessment of findings heterogeneity, the I2 test was applied in a range of 0-100% with the following explanation; 0% = no evidence of heterogeneity; 30-60% = moderate heterogeneity; and 75-100% = high heterogeneity [31]. To assess the outcomes after the negation of heterogeneous studies, a sensitivity analysis was performed [32]. Furthermore, the publication bias was analyzed by visual assessment of funnel plot symmetry [33].

## RESULTS

### Selection of studies

Figure 1 shows the PRISMA flow diagram of study selection. An electronic search of Cochrane (120), Medline (363), and EMBASE (169) databases yielded 656 articles. Hand searching of six journals published in 2021 yielded four more articles [31-34]. After removing 155 duplicated articles, 501 articles remained. Screening based on titles and abstracts yielded 17 articles that met the inclusion criteria and underwent full-text analysis (inter-reviewer agreement kappa = 0.84). The full-text assessment revealed that nine articles were accepted to include in this systematic review and analysis [10,20-23,25,26,35,36]. Thus, eight articles could not undergo meta-analysis (inter-reviewer agreement kappa = 1) because three articles [27,37,38] evaluated AgP and CP cases altogether, and two other studies [39,40] only evaluated CP patients. Three other RCTs were excluded due to not assessing the clinical parameters, having a control group not meeting the inclusion criteria, or being a review article (Appendix S1) [41-43]. Eventually, the data of nine articles were extracted and underwent qualitative and quantitative analyses by the software.

### General characteristics of the included studies

In this study, nine RCTs conducted in Iran [26], Saudi Arabia [20], Poland [35,36], Brazil [10,23,25], Turkey [22], and India [21] were evaluated (Table 1). Arwailer et al. [36], 2012, reported clinical parameters after a 3-month follow-up and then reported their findings at the 6-month follow-up in another study conducted in 2014 [35]. The number of enrolled patients ranged from a minimum of 9 to a maximum of 24, with a mean age of 27.5-37.4 years. The mean female/male percentage was 5.8-71%. All nine included studies only evaluated AgP patients. The test group of studies received aPDT following non-surgical mechanical debridement (SRP). The control group received SRP alone or along with antibiotic therapy with AMX/MET. SRP was conducted with an ultrasonic scaler [20,21,26] in some studies while hand instruments were also used in addition to an ultrasonic scaler in some other studies [22,23,36]. Only one RCT used hand instruments for SRP [10]. Andere et al. [25] reported that SRP should be performed until plaque index reaches below 20%. Among the studies in which the control group received SRP plus antibiotic therapy with 375 [40] to 500 mg [20] AMX and 250 [40] to 500 [20] mg MET 3 times a day for a total duration of 7 days, five studies reported the results at the 3-month follow-up [10,21-23,35,36], two studies reported the results at the 6-month follow-up [25,35], and one study [20] reported the results at both the 3- and 6-month follow-ups. The CAL reported outcomes varied from studies reporting the optimal efficacy of aPDT in AgP patients [20,21,23] to those not reporting any advantage over SRP alone [10,22,25,26]. Some others reported superior results in the group that received AMX/MET [35,36] (Table 1).

**TABLE 1:**
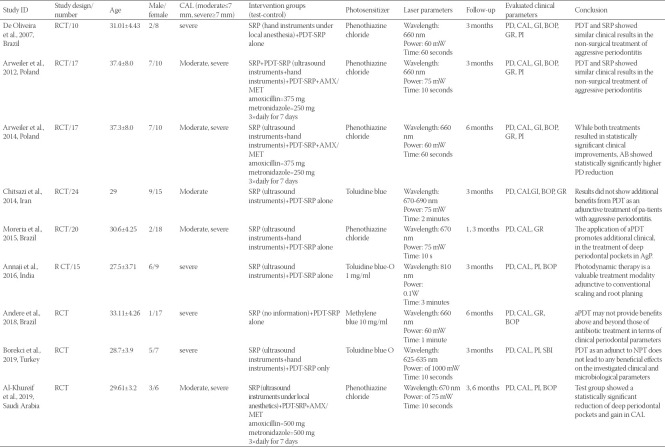
Key characteristics of included studies

### Laser parameters in the included studies

The diode laser parameters varied across the included studies as follows: Wavelength of 625-810 nm, irradiation time of 60-120 seconds, and laser power of 60-100 mW. The type of photosensitizer was phenothiazine chloride in four studies [10,20,23,36], toluidine blue in three studies [21,22,26], and methylene blue in one study. The optical fiber probe diameter ranged from 0.2 to 0.6 mm as mentioned in some studies [10,20,22,23]. The pre-irradiation time of the photosensitizer ranged from 1 minute [10,20,23,25,26] to 3 minutes [21,22,36]. The frequency of application of a PDT ranged from 1 to 4 times with daily or weekly intervals.

The energy density was not calculated in five studies. Only four studies report total energy (fluency per site), and its value ranged from 2.49 to 129 J/cm^2^ [20,22,23,25].

### Quality of clinical studies

**FIGURE 2:**
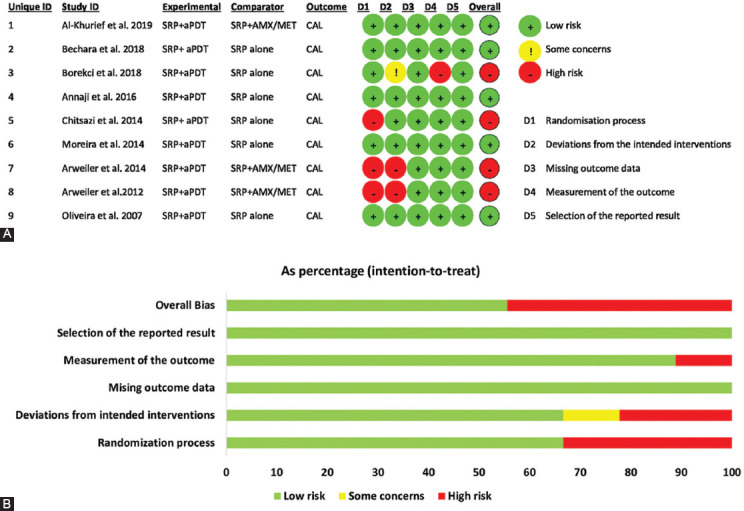
Risk of bias summary (A) and risk of bias graph (B). SRP: Scaling Root Planning, PDT: Photodynamic therapy, CAL: Clinical Attachment Loss.

The risk of bias was independently calculated for each study by two reviewers (SB and ZB) according to the recommendations of the CONSORT statement using the ROB-2 tool [44]. In this process, four studies that lacked one or more of the required parameters for qualitative assessment were classified as having a high risk of bias. Five other studies that had all the parameters were categorized as having a low risk of bias, as shown in Figure 2. In the included studies, random sequence generation was conducted with different methods. The majority of the included studies [22,23,25,35,36] used computer-generated random numbers while in two other studies [10,21], the test and control groups were randomly selected by a coin toss or flipping a coin. Two studies did not mention the randomization method [20,26].

Regarding the study design, five studies [20,22,25,35,36] had a parallel design and four studies [10,21,23,26] had a split-mouth design. The examiner was blinded to the group allocation of patients in all studies except one [22]. Three studies reported that the examiner who measured the clinical parameters was not involved in the process of examination and treatment of patients [10,25,26]. Three studies mentioned that group allocation was performed by a researcher not involved in the process of data collection and treatment of patients [20,23,25]. Furthermore, all variables mentioned in each study had no reporting bias in the analysis phase except for one study [36] that did not perform a 6-month follow-up and performed this analysis in the next study.

### The main outcome of the studies

All clinical parameters evaluated in included studies indicated the positive efficacy of aPDT + SRP for reduction of PD, clinical attachment gain, and BOP [10,20-23,25,26,35,36]; although the difference with the control group (SRP alone or SRP + AMX/MET) was not significant in most studies [25,26,36]. In CAL assessment, studies that compared aPDT with SRP + AMX/MET showed comparable efficacy of aPDT and AMX/MET therapy [20], and some others even showed that antibiotic therapy with AMX/MET was more effective than aPDT [36].

**FIGURE 3:**
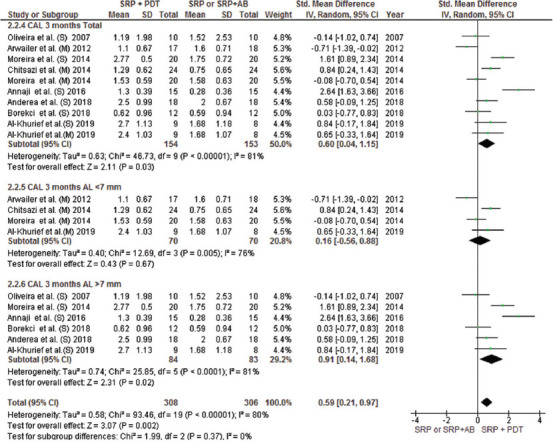
Forest plot of CAL gain at 3 months follow-up between PDT + SRP and SRP + AMX/MET groups. SRP: Scaling Root Planning, PDT: Photodynamic therapy, AL: Attachment Loss, AMX/MET: Amoxicillin/Metronidazole, CAL: Clinical Attachment Loss. \(S): Severe, (M): Moderate

**FIGURE 4:**
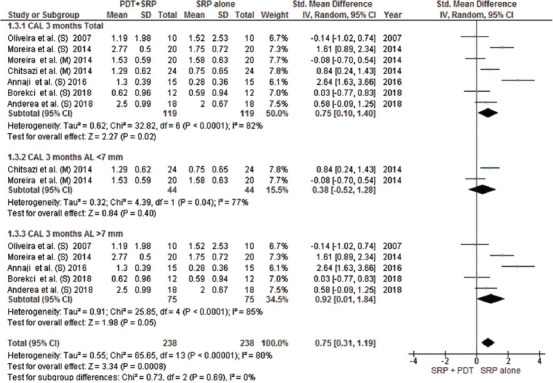
Forest plot of CAL gain at 3 months follow-up between PDT + SRP and SRP alone groups. SRP: Scaling Root Planning, PDT: Photodynamic therapy, CAL: Clinical Attachment Loss.

**FIGURE 5:**
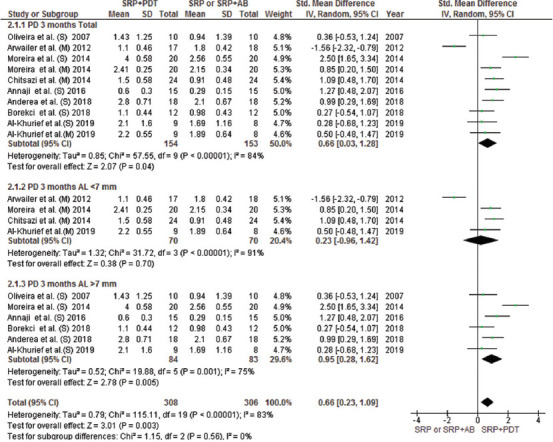
Forest plot of PD reduction at 3 months follow-up between PDT + SRP and SRP + AB or SRP alone. SRP: Scaling Root Planning, PDT: Photodynamic therapy, AB: antibiotics

**FIGURE 6:**
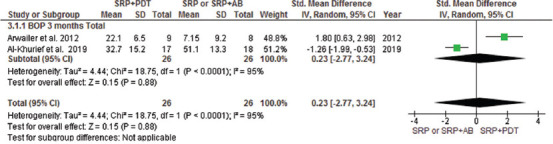
Forest plot of BOP mean reduction at 3 months follow-up between PDT + SRP and SRP + AB groups. SRP: Scaling Root Planning, PDT: Photodynamic therapy, BOP: Bleeding on probing, AB: antibiotics

This meta-analysis included all parallel design and split-mouth RCTs and compared the efficacy of aPDT with SRP alone or in combination with AMX/MET in two groups with CAL < 7 and > 7 mm. The mean difference in attachment gain at the 3-month follow-up indicated a significant difference in patients with CAL > 7 mm between the aPDT group and both SRP alone and SRP plus AMX/MET groups; however, no significant difference in CAL gain was noted in patients with CAL < 7 mm (Figures 3 and 4). The results of CAL gain at the 6-month follow-up revealed significant differences between the groups (SRP alone/aPDT and SRP+ AMX/MET/aPDT) (Appendix S2). Furthermore, the mean difference in PD reduction after 3 months was only significant in patients with CAL > 7 mm between the aPDT group and SRP alone and SRP + AMX/MET (Figure 5). However, in comparison with SRP alone, a significant difference was noted in both CAL < 7 and > 7 mm (Appendix S3). Regarding BOP, a wide variation existed among the studies in the adopted method for BOP measurement and reporting of this parameter. Resultantly, only two studies were included in this analysis, and the results indicated high heterogeneity of the studies and the absence of a significant difference among the groups (Figure 6).

### Sensitivity analysis

**FIGURE 7:**
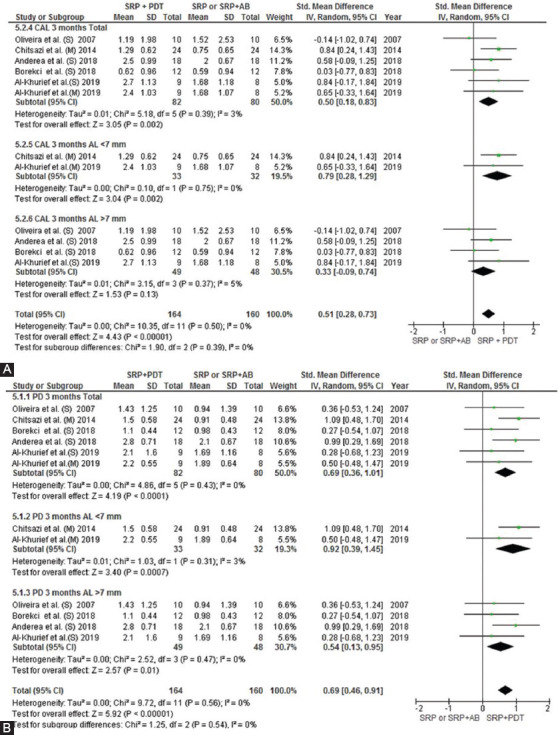
Forest plots based on sensitivity analysis showing the overall CAL gain (A) and PPD reduction (B) at 3 months without outlier studies. SRP: Scaling Root Planning, PDT: Photodynamic therapy, CAL: Clinical Attachment Loss, AB: antibiotics

A sensitivity analysis was conducted due to the noteworthy heterogeneity arising from outlier studies [21,23,35,36]. This analysis was shown only for the 3-month follow-up due to unavailability of data at 6 months in the included studies. The residual studies [10,20,22,25,26] were subjected to a sensitivity analysis which, in terms of CAL gain, disclosed statistically significant reduction (MD = 0.51 95% CI = [0.28, 0.73]; Z = 4.43 [*p* < 0.00001]) with low heterogeneity (T2 = 0.00; X2 = 10.35 [*p* = 0.5]; I2 = 0%) (Figure 7A). Improvement in PPD reduction was revealed statistically significant reduction after omitting outlier studies (MD = 0.69 95% CI = [0.46, 0.91]; Z = 5.92 [*p* = 0.56]) with no evident heterogeneity (T2 = 0.00; X2 = 9.72 (*p* < 0.00001); I2 = 0%) (Figure 7B).

### Publication bias

**FIGURE 8:**
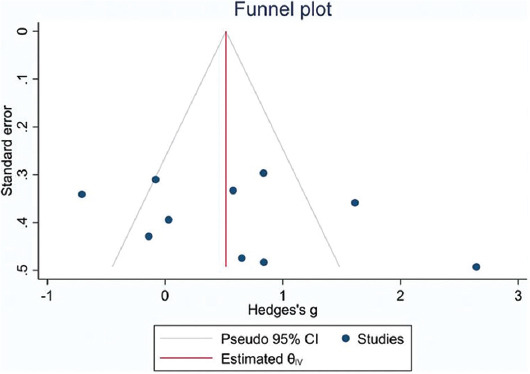
Forest plot and funnel plots for CAL gain adjusted with Trim and Fill method. Circles indicate included studies (Stata Software). CAL: Clinical attachment loss

The funnel plot of attachment gain using STATA version 16 (STATA Co., College Station, TX, USA) indicated the absence of asymmetry in the included studies (Figure 8). No asymmetry was noted when this analysis was conducted on studies that only performed SRP alone for the control group (Appendix S4). Assessment of this parameter by the Trim and Fill analysis revealed no missing study in the CAL > 7 mm group. However, in CAL < 7 mm group, one study was missed due to asymmetry (Appendices S5 and S6). Thus, the difference between the estimation of the original and adjusted effect size based on the Trim and Fill method was not significant in CAL > 7 mm group.

## DISCUSSION

The studies included in this meta-analysis evaluated the effect of aPDT as adjunctive therapy with SRP or SRP + AMX/MET on Stages II-IV Grade C molar-incisor pattern periodontitis (AgP) with CAL < and > 7 mm. In the current classification (2017), the diagnosis of periodontitis was defined in three steps: A: Staging represents severity and complexity of management. B: Extent and distribution, and C: Grading that represents evidence of risk of rapid progression and anticipated treatment response. Thus, Stages II-IV comprises patients who are diagnosed with moderate and severe (with potential for loss of the dentition) periodontitis. The rapid rate of progression represents Grade C and the molar-incisor pattern shows the distribution of the disease. The hypothesis of this study was that aPDT as adjunctive therapy in patients with CAL > 7 mm would bring about significant results, compared with those with CAL < 7 mm. However, further clinical, microbiologic, and immunological studies are required on the efficacy of aPDT in the different stages of this disease with a similar standard methodology. AgP is a relatively common inflammatory disease which can lead to early tooth loss due to CAL and extensive bone loss [45]. Antibiotic therapy and aPDT are the most commonly used non-surgical adjunctive treatments that are performed aiming to eliminate the microorganisms in hard-to-reach areas and those penetrating into the soft tissue [6,7,46]. To date, AMX/MET has been the most commonly used and most effective antibiotic regimen for AgP [47,48]. The common complications of antibiotic therapy such as the development of bacterial resistance are increasing due to widespread and negligent use of antibiotics and the consequent elimination of normal microflora. This has resulted in the application of aPDT due to its antimicrobial effects [22,23].

Several *in vitro*, *in vivo* [26,49], and animal [50] studies have shown the significant effect of aPDT with certain photosensitizers and laser energies on anaerobic microorganisms such as *Porphyromonas gingivalis* and *Aggregatibacter actinomycetemcomitans*. *P. gingivalis* is known to produce a repertoire of virulence factors that can penetrate the gingivae and cause periodontal tissue destruction directly or indirectly, by stimulation of inflammation [12]. It has also been seen that decreases in these bacterial counts are consistent with improvement of CAL gain and PD reduction [21], which are the dominant microbial species in dental plaque of patients with AgP. These microorganisms disintegrate the external membrane proteins [51,52] and produce many virulence factors that can directly or indirectly lead to the destruction of periodontal tissues by regulating host inflammatory responses [53]. Review studies and meta-analyses have shown the positive effects of aPDT on microorganisms [24,28,54-57].

With respect to the treatment outcome, all studies included in this review showed that aPDT was effective for the improvement of CAL gain and PD reduction in AgP patients; among which, only two studies reported a significant reduction in PPD and CAL parameters compared with the control group [21,23]. Two studies reported the optimal efficacy of aPDT only in deep pockets [20,23]. In the present study, aPDT along with SRP was significantly superior to the control group (SRP alone and SRP + AMX/MET) only in cases with CAL > 7 mm after 3 months. The results showed that aPDT plus SRP caused no significant improvement in clinical parameters in the short term in cases with CAL < 7 mm. In an assessment of oral hygiene instruction practiced in studies, only three out of eight studies [10,20,23] emphasized oral hygiene programs and home care instruction before mechanical treatments. Furthermore, complete debridement varied from conduction of SRP under local anesthesia [10,20,25] to debridement with ultrasonic and hand instruments in several sessions, and its continuation or discontinuation throughout the study can cause bias. Moreover, in RCTs with a control group of SRP + AMX/MET, high heterogeneity was noted due to high variation in the dosage of antibiotics (375-500 mg AMX and 250-500 mg MET). Such a high heterogeneity was also noted in the type of photosensitizer used such that phenothiazine chloride was used in five studies [10,20,23,35,36], while the remaining four studies used toluidine blue or methylene blue [21,22,25,26]. Excess photosensitizer was removed from the pockets with saline, water, or air jet after 1 [10,20,23,25,26] or 3 [22,36] minutes. Only one study [20] reported the concentration of photosensitizer used (10 mg). Thus, although the application of aPDT with 10 mg toluidine blue and methylene blue has been reported to be 100% effective in the elimination of *A. actinomycetemcomitans* *in vitro* [58], since the concentration of residual photosensitizer after rinsing the pocket with different techniques is not known, the concentration of reactive oxygen species for the elimination of pathogens involved in AgP cannot be assessed. Furthermore, the energy used per square millimeter varied in different studies. Five studies were not report this information [10,21,26,35,36]. In two studies, fluency per site was reported at 2.49 J/cm^2^ [20,23]. Although, two studies used different fluency per site (20 [22] and 129 [25] J/cm^2^) in their studies.

Another reason responsible for high heterogeneity is the high variation in the frequency of application of aPDT with variable intervals. aPDT is recommended to be repeated over several sessions because it has been shown that SRP has a short-term effect, and recolonization of pocket occurs after 3 weeks [59]. Thus, aPDT should be preferably repeated for several sessions [54] to prevent pocket recolonization. It should be noted that the majority of studies included in this meta-analysis did not report the degree of improvement in clinical parameters separately after each treatment session. Only one study [21] compared the treatment results after the first and fourth treatment sessions. Furthermore, the three studies [20,21,23] that performed four sessions of PDT reported controversial results; however, they all reported maximum efficacy of aPDT in deep pockets. One study [20] reported that aPDT had no advantage over AMX/MET in the improvement of clinical parameters. Moreover, improvement of clinical parameters was not reported in different levels of CAL with specific and standardized control groups in the included studies. As a result, due to the high heterogeneity, sensitivity analysis was performed and the outline studies in visual inspection of forest plot analysis were omitted until low heterogeneity was achieved. This sensitivity analysis shows a significant difference in the CAL and PPD parameters of the PDT + SRP application versus SRP alone or with AMX/MET (Figure 7). Thus, RCTs with the same reproducible methodology are required on different levels of CAL with longer follow-ups to find the most effective treatment based on the type and number of plaque microorganisms. In general, high heterogeneity in methodologies such as plaque control methods, oral hygiene instructions provided to patients, technique and frequency of SRP sessions, laser parameters, output energy per surface unit (J/cm^2^), frequency of aPDT sessions, and type of photosensitizer in RCTs also cause bias and prevent achieving reliable results. Moreover, a recent study [60] has identified that certain wavelengths should be used with blue photosensitizers, and there is no photodynamic reaction at wavelengths above 800 nm (infrared) with the blue type of PS (toluidine blue and methylene blue). Hence, blue photosensitizers are used just with 635-660 nm wavelengths. Therefore, the RCTs that have used wavelengths above 800 nm with blue PS can cause bias in the systematic review and meta-analysis. Due to this, Annanji et al. (2016) study, which treated the patients with aPDT (wavelength 810 nm, PS: toluidine blue), was omitted in sensitivity analysis and the outcome shows a significant difference in the application of PDT in terms of CAL parameter.

However, standardized treatments customized based on disease severity can estimate the necessity of application of this treatment modality and prevent its unnecessary prescription.

Assessment of attachment gain and PD reduction can help determine the necessity of conducting of aPDT, and a comparison between the effect sizes of different meta-analyses can greatly aid in designing a successful treatment plan.

**FIGURE 9:**
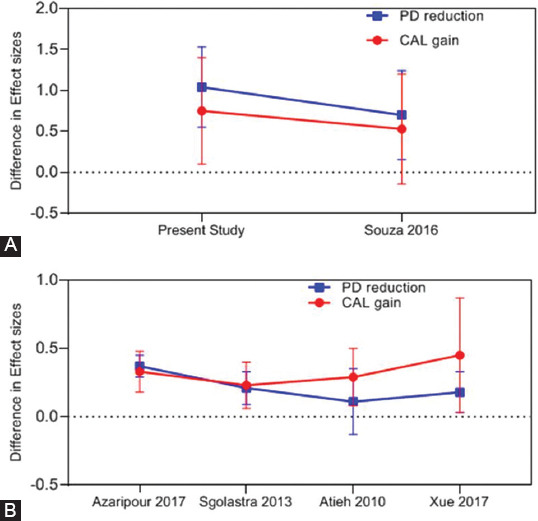
Effect size comparison of different meta-analysis studies in (A): CP patients (PDT+SRP vs. SRP) and (B) AgP patient (PDT + SRP vs. SRP alone).PD: Probing Depth, CAL: Clinical Attachment Loss, PDT: Photodynamic therapy, Agp: Aggressive periodontitis, SRP: Scaling Root Planning.

The effect of aPDT versus SRP alone (as the control group) on AgP is important in terms of the effect size of attachment gain and PD reduction in meta-analyses, as shown in Figure 9A. Accordingly, the present meta-analysis indicated greater improvement of clinical parameters following PDT compared with other meta-analyses. Furthermore, a parallel assessment of meta-analyses on clinical parameters in CP patients revealed that despite the differences in the results of analyses (Figure 9B), better performance of aPDT was noted in CP compared with AgP (Figure 9B). The reason appears to be the greater presence of local factors such as calculus and dental plaque in CP, which are the main causes of the development of CP [61] and can be well eliminated by mechanical treatments. In other words, it may be stated that mechanical treatments in AgP cannot easily and completely remove the microorganisms and lead to acceptable recovery due to the presence of numerous risk factors, extensive CAL, and penetration of invading bacteria into deep tissues. Thus, aPDT causes greater improvement in AgP patients due to its antibacterial activity against the causative microorganisms lodged in deep tissues.

**FIGURE 10:**
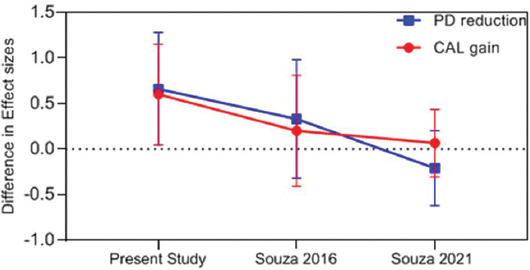
Effect size comparison of different meta-analysis studies in AgP patients (PDT + SRP vs. SRP + AMX/MET). PD: Probing Depth, CAL: Clinical Attachment Loss, PDT: Photodynamic therapy, AMX/MET: Amoxicillin/Metronidazole, SRP: Scaling Root Planning.

In some meta-analyses, treatment with AMX/MET was performed combined with SRP for the control group, as shown in Figure 10. The results indicated that regarding the effect size of attachment gain, aPDT + SRP caused a greater improvement by 50-90% compared with SRP + AMX/MET in the present study compared with other meta-analyses. This improvement can be due to a higher number of more recent studies included in the present meta-analysis. Furthermore, in the majority of studies, the level of attachment gain and PD reduction in the aPDT group was comparable to those in treatment with AMX/MET except for the study by Souza et al. (2021) [28] that showed that antibiotic therapy with AMX/MET was more successful than aPDT.

**FIGURE 11:**
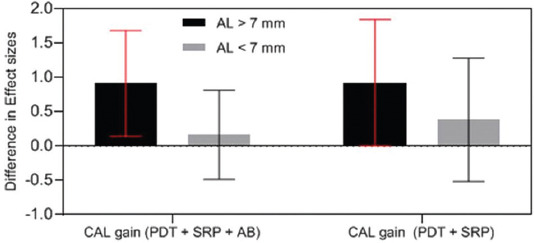
Effect size comparison of CAL gain of AL> 7 mm and AL < 7 mm with different control groups. CAL: Clinical Attachment Loss, AL: Attachment Loss, AB: Antibiotic, SRP: Scaling Root Planning, PDT: Photodynamic therapy

Although the present results were generally in line with the findings of recently published review articles [28,54] regarding the positive effect of PDT on AgP versus SRP or AMX/MET as adjunctive treatments, a more precise analysis of the effect size of attachment gain in the SRP alone and SRP + AMX/MET control groups in meta-analyses indicated 80% and 60% efficacy for aPDT, respectively, only in CAL > 7 mm. Furthermore, the level of improvement caused by aPDT in cases with CAL > 7 mm was almost the same in both control groups. In cases with CAL < 7 mm, AMX/MET did not cause a significant improvement in this parameter compared with SRP alone. In other words, aPDT is an effective treatment for cases of AgP with CAL > 7 mm compared with mechanical treatment alone (Figure 11). Furthermore, since AMX/MET is more effective in cases with extensive CAL and deep pockets, the greatest improvement and microorganism reduction occur in cases with CAL > 7 mm.

Since the effect of different confounding factors was not adjusted, and different grades of disease were not evaluated in this study, the results of the analyses are not 100% reliable. However, it should be kept in mind that adjunctive treatments should be adopted only in a severe form of the disease for the elimination of microorganisms in hard-to-reach areas. Furthermore, considering the possible side effects such as eye injury in case of not correctly using protective eyeglasses, thermogenesis, and periodontal tissue injury due to the use of chemicals in effective concentrations [17], negligent use of such modalities in mild cases should be avoided due to the absence of conclusive evidence.

This study had some limitations such as the small number, and high heterogeneity of the included studies such that in qualitative analysis, only two studies had a low risk of bias. Furthermore, due to the limitations of RCTs in the assessment of different levels of CAL, the mean attachment loss reported in studies was used to categorize disease severity based on CAL < 7 mm and > 7 mm.

## CONCLUSION

Analysis of included studies indicated a significant difference in clinical attachment gain in patients with CAL > 7 mm between the aPDT group and the SRP alone and SRP + AMX/MET control groups. However, this difference was not significant in patients with CAL < 7 mm.

Despite the limitations within this meta-analysis, the aPDT was suggested as adjunctive therapy in the treatment of Stages II-IV, Grade C molar-incisor pattern periodontitis with CAL > 7 mm, although improvement of clinical parameters in the patients with CAL< 7 mm remains debatable.

## Figures and Tables

**APPENDIX S1:**
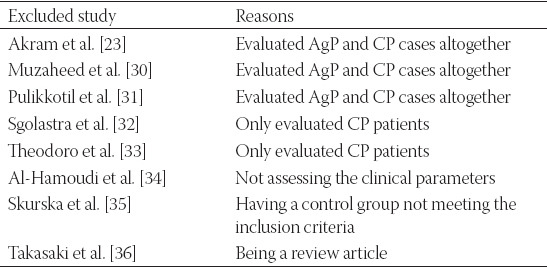
List of reasons for exclusion of articles in the stage of full-text assessment

**APPENDIX S2:**
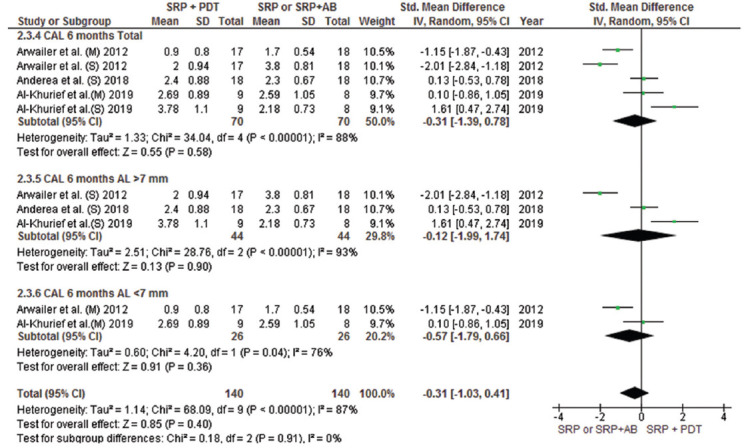
Forest plot of CAL gain at 6 months follow-up between PDT+SRP and SRP+ AB groups. SRP: Scaling Root Planning, PDT: Photodynamic therapy, CAL: Clinical Attachment Loss, AB: Antibiotics.

**APPENDIX S3:**
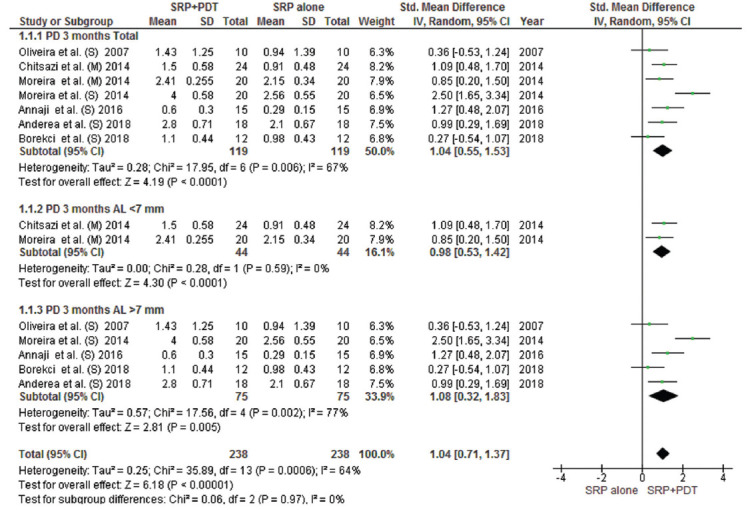
Forest plot of PD reduction at 3 months follow-up between PDT+SRP and SRP alone. SRP: Scaling Root Planning, PDT: Photodynamic therapy, AL: Attachment loss.

**APPENDIX S4:**
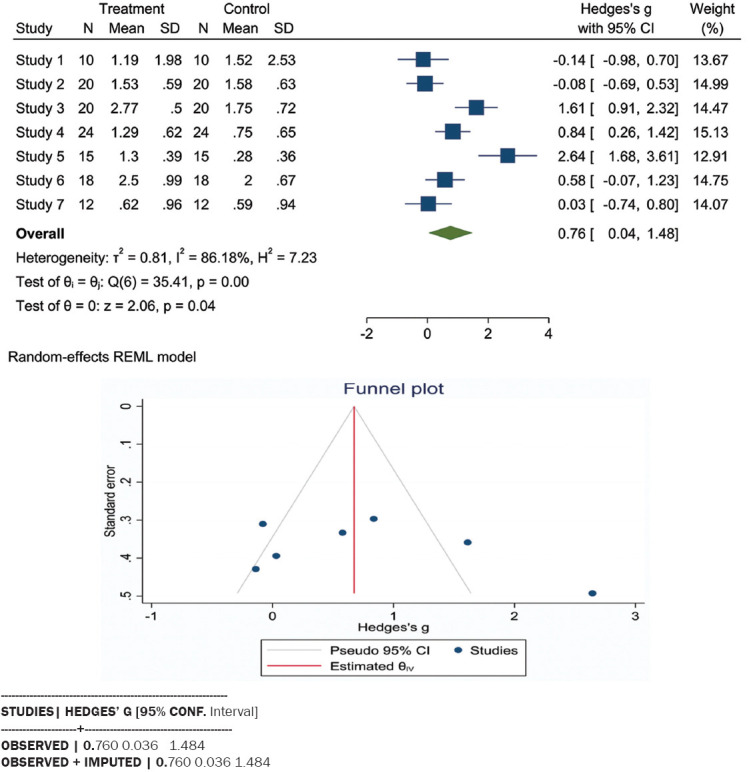
Forest plot of CAL gain, funnel plot, and Trim and Fill method at 3 months follow-up between PDT +SRP and SRP alone (total), SRP: Scaling Root Planning, PDT: Photodynamic therapy, CAL: Clinical attachment loss.

**APPENDIX S5:**
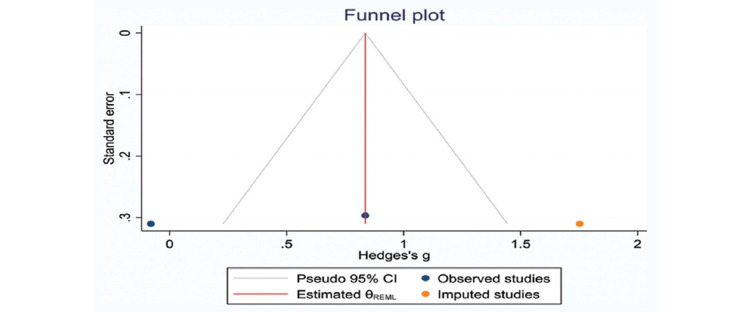
Forest plot of CAL gain, funnel plot, and Trim and Fill method at 3 months follow-up between PDT +SRP and SRP alone groups in attachment loss <7 mm (Stata Software).

**APPENDIX S6:**
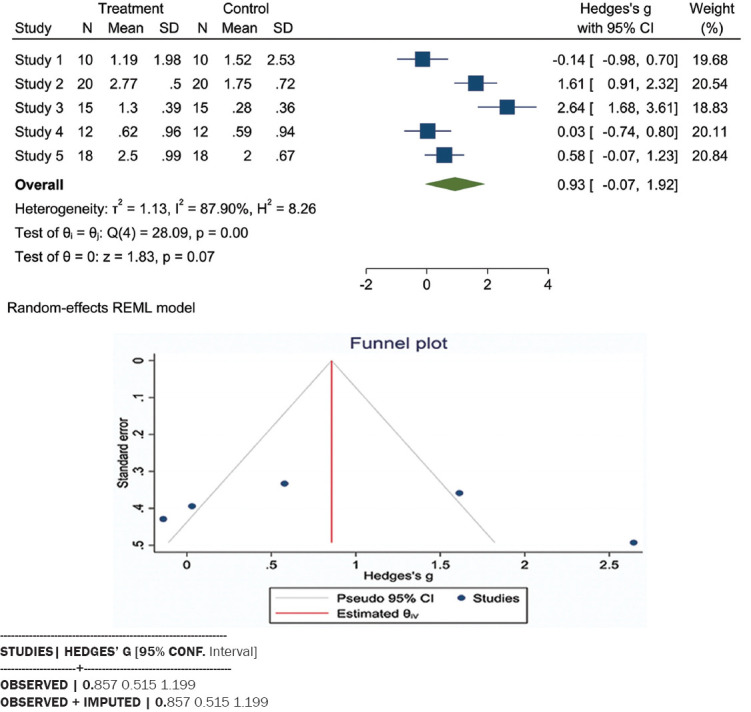
Forest plot of CAL gain, funnel plot, and Trim and Fill method at 3 months follow-up between PDT + SRP and SRP alone groups in attachment loss >7 mm (Stata Software). SRP: Scaling Root Planning, PDT: Photodynamic therapy, CAL: Clinical attachment loss.
